# Microdialysis for chronic exertional compartment syndrome: a pilot study 

**DOI:** 10.1186/s13102-021-00245-9

**Published:** 2021-03-05

**Authors:** Heinz Lohrer, Jochen Klein, Tanja Nauck, Tobias Schönberg

**Affiliations:** 1 ESN – European SportsCare Network, Borsigstrasse 2, 65205 Wiesbaden, Germany; 2 Department of Sport and Sport Science, University of Freiburg, Freiburg, Germany; 3 Institute for Pharmacology and Clinical Pharmacy, Goethe-Universität Frankfurt, Frankfurt, Germany; 4 Solupharm, Melsungen, Germany

**Keywords:** Exercise-induced leg pain (EILP), Chronic exertional compartment syndrome (CECS), Microdialysis, Muscle metabolism, Leg, Overload-induced injury

## Abstract

**Background:**

Diagnosing chronic exertional compartment syndrome (CECS) is still a challenge. An increase in intramuscular pressure during and following exercise is accepted as the diagnostic standard. However, neither the methods used nor the interpretation of the obtained results are sufficiently standardized.

**Methods:**

In the present pilot study, the metabolic state of CECS patients was investigated using microdialysis. We hypothesized that there was no difference in intramuscular concentrations of glucose, lactate, glutamate, and glycerol before and after exercise (H1_0_) or between patients suffering from CECS and healthy control subjects (H2_0_). This study was designed as an explorative case-control study (level of evidence III). Twelve patients suffering from CECS of the lower leg and six matched asymptomatic control subjects underwent microdialysis in the anterior (*n* = 7) or deep posterior compartment (*n* = 11) of the leg. Following ultrasound-guided insertion of the microdialysis catheters, 10-minute fractions of the dialysates were collected first during rest and then following fatigue- or pain-induced discontinuation of exercise. Dialysates were analysed for lactate, glucose, glutamate, and glycerol concentrations 6 × 10 min before and 6 × 10 min after exercise.

**Results:**

Exercise-induced increases in lactate, glutamate, and glycerol concentrations were detected in both CECS patients and control subjects (all *p* < 0.001). No differences between CECS patients and control subjects were found by comparing the intramuscular glucose, lactate, glutamate, and glycerol concentrations at rest and following exercise (all *p* > 0.05).

**Conclusions:**

We found exercise-induced increases in the lactate, glutamate, and glycerol levels in skeletal muscle. However, the metabolic changes did not differentiate CECS patients from healthy subjects.

**Trial registration:**

The registration trial number is DRKS00021589 on DRKS. ‘Retrospectively registered’. Date of registration: April 4, 2020.

## Background

Chronic exertional compartment syndrome (CECS) is the most common entity under the “umbrella” term of exercise-induced leg pain. Per definition, non-traumatic activity-related pain in or over the involved compartment starts after an individually reproducible time or intensity of activity or running distance and increases until the pain forces the patient to stop the activity inducing the pain [1, 2]. Within minutes of rest or interruption, the symptoms subside. CECS predominantly involves the anterior and/or posteromedial leg compartments of runners.

The history reveals the typical clinical constellation of CECS and physical examinations present unremarkable findings. Clinical diagnosis therefore relies on excluding any intervening pathologies [1, 2]. An accurate assessment is essential for assigning further treatment. Most authors favour operative compartment release when conservative therapy fails [2].

Based on suspicion of an exercise-induced history, compartment pressure measurements became the standard for confirming a CECS diagnosis. However, clear-cut and generally accepted diagnostic criteria obtained from pressure measurements are not established so far due to different technologies, validities of the measurement devices, and load protocols [3–6]. Specifically, deep posterior compartment pressure measurements were found to be unsafe and therefore unnecessary due to “uncertainty of needle placement and potential for neurovascular injury” [7].

Surgery can effectively be performed even in patients with normal or slightly increased intramuscular pressure. Therefore, the need for pressure measurements to diagnose CECS of the lower leg is questioned [8–10]. The diagnostic value of intracompartmental pressure measurement and near infrared spectroscopy in CECS were reported to be equivalent, while MRI was considered to be less suitable [11]. Near infrared spectroscopy demonstrated a greater relative de-oxygenation during exercise as well as delayed re-oxygenation after exercise in CECS patients, supporting an ischaemic aetiology of the condition [12].

Microdialysis is a minimally invasive diagnostic procedure [13, 14]. A semipermeable membrane continuously extracts freely diffusible, water-soluble molecules from the extracellular space of the investigated tissue. In recent years, microdialysis is being increasingly used in clinical intensive care and for in vivo research [14]. “During microdialysis, analytes pass through a semipermeable membrane from the extracellular fluid into a perfusate that is collected over a predetermined time and volume” [14]. Markers of energy metabolism (glucose, lactate, glutamate) and cell damage (glycerol) can be analysed [13–16].

Microdialysis has recently been introduced into sports orthopaedics to study and understand the translation of mechanical load applied to specific tissues during biological adaptation processes or pathologic reactions [15, 17–20].

The purpose of this study was to investigate if microdialysis could be an objective tool to differentiate patients with anterior or deep posterior leg CECS from uninjured persons. The H1_0_ hypothesis was that there is no difference in the concentration of metabolic markers of energy metabolism (glucose, lactate, glutamate) and cell damage (glycerol) in the dialysates of CECS patients and control subjects before and following exercise. The H2_0_ hypothesis was that there is no difference in the concentration of these markers between control subjects and patients suffering from CECS in the anterior or deep posterior leg compartment before and following exercise.

## Methods

This explorative pilot study was designed as a case-control study (level of evidence III). The local ethics committee approved the study (FF 33/2009). The registration trial number is DRKS00021589 on DRKS. ‘Retrospectively registered’. Date of registration: April 4, 2020.

### Patients and control group participants

We recruited patients and control group participants from the orthopaedic clinic of our sports medicine centre. The control group participants were selected to resemble the patient group with respect to anthropometric data and sport/running behaviours (all *p* > 0.05; Table 1). Eligible participants were screened according to our inclusion and exclusion criteria. Selected participants received detailed oral and written information about the study project, design, and operational aspects of the study and provided written informed consent.

**Table 1:** Anthropometrics, preferred sports, running duration within the experiment (experimental load), and numbers of analysed compartments for the tested groups

	CECS Patients	Controls	*p*-value
*N*	12	6	n.a.
Age [years]	23 ± 7.6 (15–37)	21 ± 1.0 (19–24)	0.596
Height [cm]	177 ± 8 (158–185)	182 ± 10 (198–167)	0.304
Weight [kg]	67.9 ± 12.0 (50.0–100.0)	67.2 ± 9.3 (54.0–77.0)	0.908
BMI [kg/m^2^]	21.7 ± 3.0 (17.9–29.9)	20.2 ± 0.6 (19.4–21.1)	0.300
Sport			n.a.
• Running	8	3	
• Swimming	2	1	
• Volleyball	0	2	
• Rowing	1	0	
• Basketball	1	0	
Experimental load [min]	36 ± 18.8 (15–90)	29 ± 4.2 (25–35)	0.442
Analysed Compartments [n]			n.a.
• Females	7	4	
• Males	5	3	
• Anterior	4	3	
• Deep posterior	8	3	
• Right/left	5/7	3/3	

*N.a.* not applicable

Participants between 15 and 50 years of age were included in either group. All participants had to perform recreational or competitive sport activity on a regular basis (≥2×/week and/or ≥ 2 h/week). Patients were included in the patient group if CECS was diagnosed from a typical history and unremarkable physical examination. Uni- or bilateral anterior and deep posterior CECS with a spontaneous, non-traumatic onset were accepted. There was no preference for anterior or posterior CECS given from the study protocol. During the study we recruited 12 CECS patients for further analysis. Eight of them were deep posterior and four were anterior (Table 1). Specifically, a minimum of 12 weeks history of running-induced pain was required, which reproducibly irradiated over more than 10 cm of the deep posterior or anterior leg compartment. Finally, the increasing pain had to force the patient to interrupt the running activity. After cessation of the inducing activity, the symptoms had to be relieved completely within less than 5 min. For the control group, no history of exercise-induced leg pain and an unremarkable physical exam were used to exclude CECS.

Exclusion criteria for both groups were: exercise induced leg pain (EILP) different from CECS, such as bone stress injuries, pain of osteo-fascial origin, particularly medial tibial stress syndrome, pain of muscular origin, pain due to nerve entrapment, and pain due to a temporary vascular compromise [2]. We excluded non-athletes, persons with abnormal alignment of the lower extremity, uncertain compliance, acute or degenerative spine diseases, systemic diseases (e.g., diabetes), and persons who participated in other clinical studies up to 1 y ago. We also excluded patients with acute or traumatic onset of symptoms or with previous leg surgery.

**Fig. 1:**
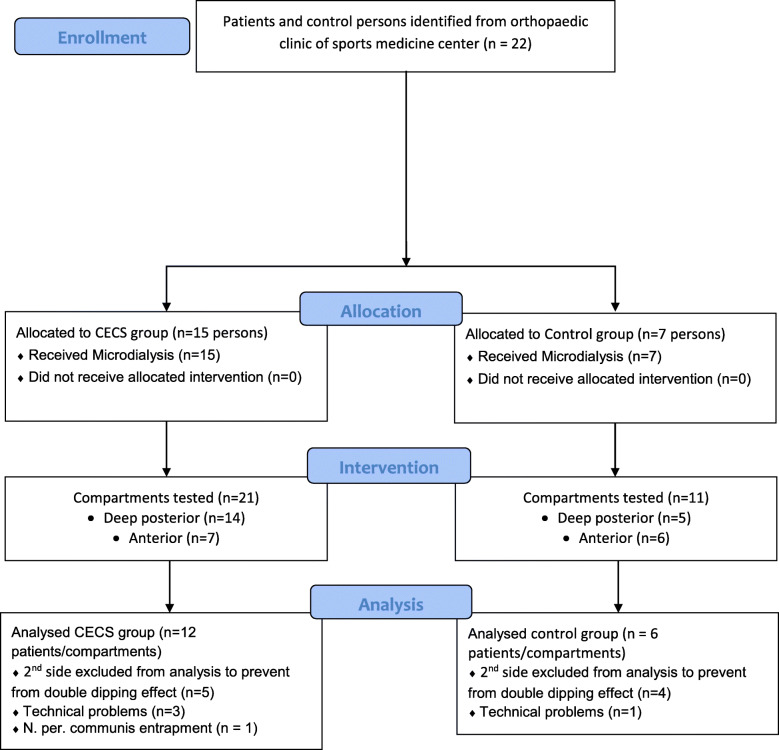
Flow chart [22] for participants’ inclusion in the study

Only one compartment per leg was analysed. When CECS occurred bilaterally, we tested both sides. To avoid a ‘double-dipping effect’ [21], we chose the more painful leg of the patients for further analysis (Fig. 1) [22]. For the control persons, the side to evaluate was chosen randomly.

### Microdialysis and experimental procedure

With the tested person lying supine, the microdialysis probe was inserted under ultrasound guidance into the middle of the respective muscle belly. According to the manufacturer’s instructions and under sterile conditions, a sterile, single use microdialysis probe (CMA 63, M Dialysis AB, Stockholm, Sweden) was inserted into the muscle parallel to the fibres with a splitable introducer. The shaft was 40 × 0.9 mm and the membrane length was 30 mm. The membrane cut-off was approximately 20,000 Da. The microdialysis catheter was fixed on the leg with tape. The microdialysis catheter’s inlet tube was connected to a microdialysis pump. The outlet tube ended with a microvial holder where the sample was collected into small microvials. The probes were perfused with sterile Ringer solution at a flow rate of 2 μl/min by means of a precision infusion pump (CMA 100, CMA Microdialysis, Stockholm, Sweden). Sampling was performed for 6–12 × 10 min. Then, the whole microdialysis system was removed and the point of insertion was dressed with a sterile tape. The patients then ran in the adjacent forest at his/her preferred speed. The patients returned when the typical CECS pain forced them to discontinue. Control persons ran a distance of 5 km with an exhaustive speed. Then, another microdialysis measurement was commenced within 10 min and sampling was performed for another 6–8 × 10 min.

### Dialysate analysis

Samples in the microvials (10–20 μL) were initially frozen at − 80 degrees C and were later analysed using micro-analysers as previously described [13, 15]. Glucose, glutamate and lactate concentrations represent energy metabolism while glycerol represents a mediator of cell damage. Samples obtained 6 × 10 min before and after the exercise load, respectively, were analysed and included for further statistical processing. In the first step, means and standard deviations were calculated for each pre- and post-load 10 min sampling period. We defined a ‘rest’ phase including the 6 × 10 min samples before exercise (first microdialysis probe), a ‘peak’ phase including the initial two samples after exercise and implantation of the second microdialysis probe, and a ‘recovery’ phase as the 4 × 10 min measurements following the ‘peak’. For the longitudinal pre- and post-exercise analyses within the CECS and control group, respectively, the 6 × 10 min ‘rest’ dialysates were compared with the ‘peak’ dialysates after exercise and implantation of the second microdialysis probe. The respective results were averaged within the groups.

### Statistical analysis

Anthropometric data and running duration between groups were compared by t-tests for independent samples. Repeated measures two-way ANOVA (Software: GraphPad® Prism 4.0, San Diego, CA) was performed to identify between-group differences (CECS vs. control), time effects, and group × time interaction effects. There were 22/1056 (2.1%) values missing within all individual data sets. For further statistical analysis, these values were filled in by interpolation. One-way ANOVA was used to test for differences between rest, peak, and recovery phases. For the determination of statistical power, we used the eq. *N* = 2 SD^2^ × power index / delta^2^. We expected standard deviations of 25% in each group, and we aimed to find significantly different values (*p* < 0.05) at differences of 30% between healthy subjects and CECS patients. We respectively calculated the required number of participants as 8.61 patients per group. With 6 healthy volunteers and 12 patients, our study has a power of approximately 80%. The power index of 6.2 was taken from Harvey Motulsky’s textbook “Intuitive Biostatistics” [23].

## Results

**Fig. 2:**
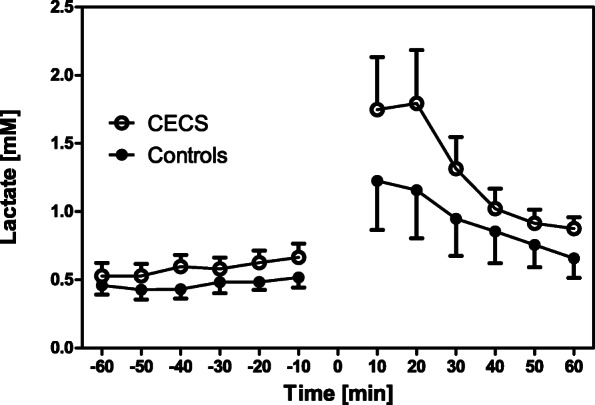
Lactate concentrations in the CECS and control group dialysates pre and post load. Mean concentrations ± standard deviations for the respective 10 min sampling periods. Statistical comparison (two-way ANOVA for repeated measurements): F_1,16_ = 1.52; *p* = 0.24. The exercise phase is indicated by 0

**Fig. 3:**
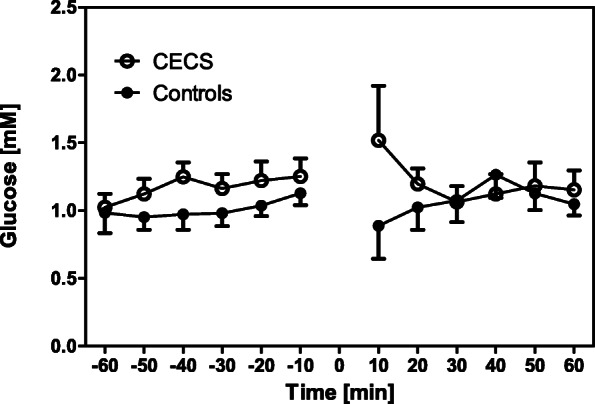
Glucose concentrations in the CECS and control group dialysates pre and post load. Mean concentrations ± standard deviations for the respective 10 min sampling periods. Statistical comparison (two-way ANOVA for repeated measurements): F_1,16_ = 0.91; *p* = 0.35. The exercise phase is indicated by 0

**Fig. 4:**
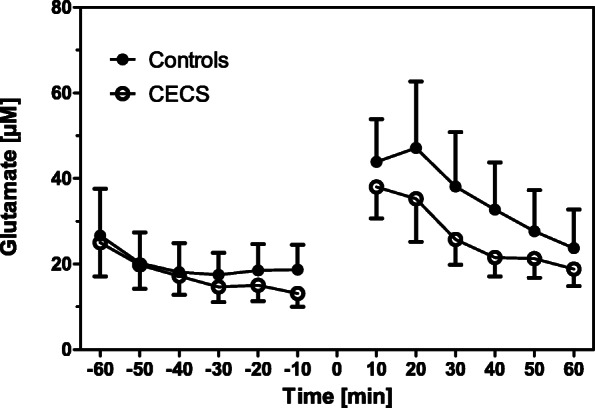
Glutamate concentrations in the CECS and control group dialysates pre and post load. Mean concentrations ± standard deviations for the respective 10 min sampling periods. Statistical comparison (two-way ANOVA for repeated measurements): F_1,16_ = 0.46; *p* = 0.51. The exercise phase is indicated by 0

**Fig. 5:**
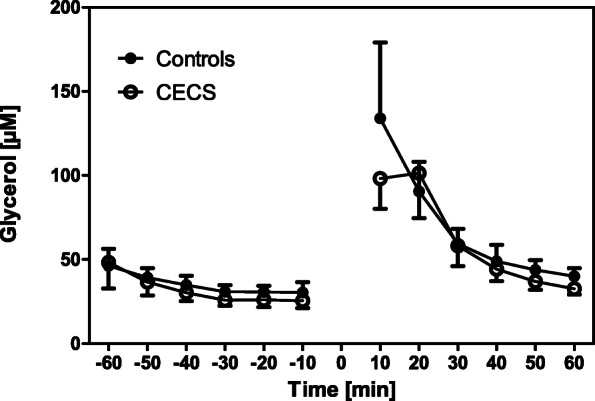
Glycerol concentrations in the CECS and control group dialysates pre and post load. Mean concentrations ± standard deviations for the respective 10 min sampling periods. Statistical comparison (two-way ANOVA for repeated measurements): F_1,16_ = 0.23; *p* = 0.64. The exercise phase is indicated by 0

There were no between-group differences for the anthropometric data (all *p* > 0.05; Table 1). Two-way ANOVA for repeated measurements found no differences between the time courses of metabolites between the control and CECS groups (lactate, *p* = 0.24; glucose, *p* = 0.35; glutamate, *p* = 0.51; glycerol, *p* = 0.64. Figures 2, 3, 4, 5). Time effects were significant for lactate, glutamate, and glycerol. There were no group × time interaction effects for any metabolites.

**Fig. 6:**
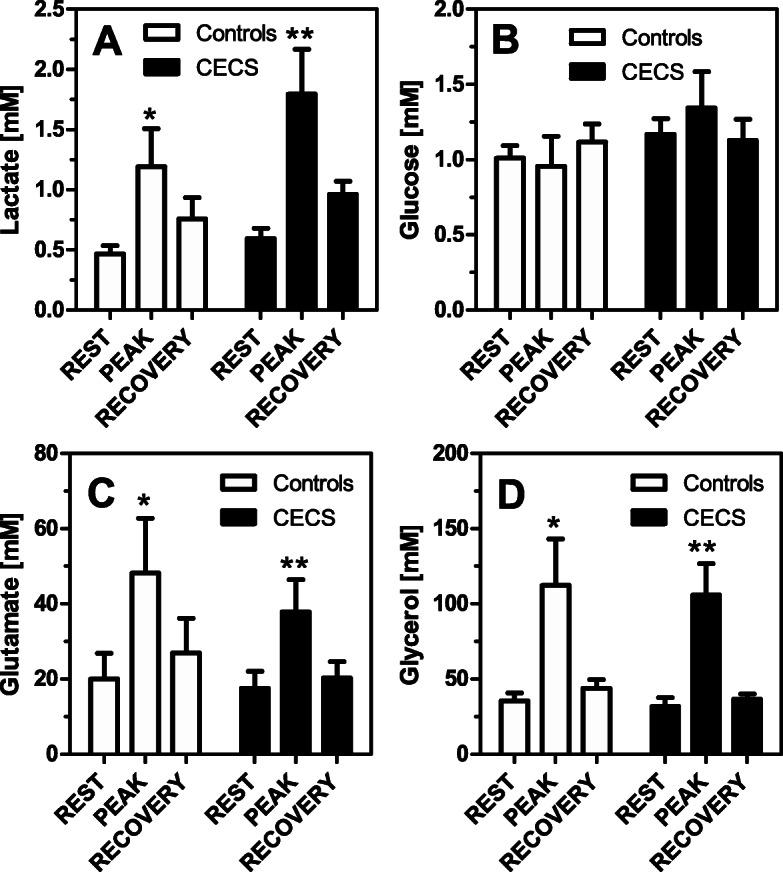
Lactate, glucose, glutamate, and glycerol concentrations in the CECS and control group dialysates at 6 × 10 min of rest, 2 × 10 min following exhaustive exercise (‘peak’), and 4 × 10 min of ‘recovery’. One-way ANOVA. * = *p* < 0.05; ** = *p* < 0.01

One-way ANOVA revealed that compared to ‘rest’, lactate, glutamate, and glycerol concentrations increased in the ‘peak’ phase following exercise in both groups (*p* = 0.01 to 0.05, respectively). Glucose levels were unaffected by exercise (*p* > 0.05; Fig. 6).

## Discussion

This explorative pilot study is the first investigation to test if microdialysis is able to demonstrate differences in muscle metabolism in leg compartments before and following exercise. We analysed markers of the energy metabolism under CECS conditions. This idea is derived from the hypothesis, that lactate and further markers of energy metabolism accumulate more in the CECS muscle due to fascial compression. H1_0_ is rejected by our findings. Compared to ‘rest’, mean post-exercise metabolite (‘peak’) concentrations increased significantly for lactate, glutamate, and glycerol in the CECS and control groups (all *p* < 0.001; Fig. 6). With the available numbers H2_0_ is confirmed, because microdialysis was unable to differentiate CECS from uninjured compartments. This means that between-group differences were not detected for the tested metabolites.

Stable metabolite concentrations with low standard deviations existed during ‘rest’. This behaviour indicates that the microdialysis procedure was valid and reproducible in our experiments. After exercise, mean lactate, glutamate, and glycerol concentrations in both groups increased when compared with ‘rest’, and they recovered after a post-exercise peak during the recovery phase (Figs. 2, 3, 4, 5, 6). This increase was insignificantly higher in the CECS group.

These results are in line with previously published hypotheses regarding the aetiology of CECS. Most authors assume that CECS develops from a mismatch of a small compartment, a bulky muscle, and a stiff fascia. The resulting hypertension may restrict muscle perfusion within the compartment, leading to pressure-induced ischaemic pain and muscle cell damage [2, 11, 12, 24, 25]. Little research has elucidated the metabolic conditions in CECS. In percutaneous needle biopsies from eight healthy individuals, lactate was elevated six and 1.2 times in the anterior and deep posterior muscle immediately (< 2 min) after exhausting isokinetic dynamometer exercise [24]. Corresponding intramuscular pressure measurements demonstrated a two-fold elevated mean pressure immediately (0 min) after exercise in the anterior compartments. One minute after exercise, the anterior compartment pressure decreased to the pre-exercise level [24].

The anterior CECS was also studied by muscle biopsy and intracompartmental pressure measurements at rest and immediately after isometric exercise before and after fasciotomy [25]. A significant lactate concentration increase in these muscle biopsies was detected after exercise. Ten minutes to 2 h after exercise, the intracompartmental pressure returned to resting values and the pressure half-value period was 6 ± 3 min [25]. More recently, a reduced microcirculation capacity was found in biopsies from anterior CECS patients [26]. In the CECS patients, this reduced microcirculation could be responsible for reduced mitochondrial oxidative activity, resulting in a lower potential for aerobic metabolism [26].

Glutamate was proposed as an excitatory mediator of pain [27]. In two small controlled cohorts, intratendineous glutamate levels during rest were higher in four symptomatic extensor carpi radialis brevis (tennis elbow) [20] and four Achilles tendinopathy [19] tendons. In contrast, the findings of the present study do not support glutamate as a neurotransmitter responsible for pain generation in CECS. In fact, high intracellular glutamate levels in skeletal muscle are explained by uptake from the blood, protein (myosin) breakdown and transamination [16]. Interestingly, post-exercise concentrations of glutamate were lower in the CECS group by tendency (Table 2; Fig. 4). In principle, a higher level of glutamate could be pain induced. However, it is more likely that it instead indicates a higher protein degradation activity. Whether this glutamate concentration increase in healthy persons is real or if there is a principally different behavior between tendons and muscles needs to be clarified in future studies.

**Table 2:** Mean values and paired-samples t-test comparisons for 6 × 10 min dialysates between pre-exercise (rest) and post-exercise (peak + recovery) for the CECS and control group

	Rest [mmol/l]	Post-exercise (Peak + recovery) [mmol/l]	***p***
**CECS**			
• Lactate	0.59	1.28	**< 0.001**
• Glucose	1.17	1.21	0.318
• Glutamate	17.58	26.99	**< 0.001**
• Glycerol	31.71	61.18	**< 0.001**
**Control**			
• Lactate	0.47	0.93	**< 0.001**
• Glucose	1.01	1.07	0.196
• Glutamate	19.96	36.42	**< 0.001**
• Glycerol	35.41	69.44	**< 0.001**

Significant findings are bold

Glycerol concentration during the experiment was very similar in both groups. Glycerol is formed by the breakdown of membrane phospholipids, which occurs during exercise. Therefore, abnormal muscle damage in CECS patients is not supported by our results.

We should consider several limitations of this study. First, the numbers within the two groups are low and therefore the resulting power of the study to significantly differentiate CECS patients from uninjured persons is low. However, rigorous inclusion and exclusion criteria produced well-defined groups for comparison. In our experiments, there was a delay of approximately 10 min between the end of the load and the completion of the insertion of the second microdialysis probe. This delay could be too long to sufficiently catch the initial metabolite peak. A continuous measurement with the same catheter could avoid that flaw, but so far, the microdialysis system cannot be applied to a working muscle.

Another weakness is that CECS diagnosis and allocation to the control group relied only on history and unremarkable findings by physical examination. An objective intracompartmental pressure measurement was not performed. Additionally, the running distances to pain induced cessation were relatively long. This may indicate a low degree of CECS with lower differences when compared to healthy persons. However, all patients presented to the clinic with substantial complaints. Due to ethical reasons, we performed no simultaneous intra-compartmental pressure measurements, which is frequently described as standard for confirming CECS [28]. Strong evidence from recent research, however, questions the value of intra-compartmental pressure measurements to effectively diagnose CECS [2, 6, 10, 11, 29, 30]. Previous studies demonstrated that the indication for operative compartment release could be based on history and clinical findings alone [8, 30]. In a previous study, the clinical CECS suspicion was retrospectively confirmed in 93.3%, while pressure measurements had a sensitivity of 77% [11].

Finally, the literature discusses an overlap between deep posterior CECS and medial tibial stress syndrome, which is defined as a bone or fascial stress injury and is not associated with hypertension in the deep posterior compartment [31–33]. With the available numbers, the large standard deviations in the initial post-exercise phase prevented the data from reaching the significance level. Therefore, additional similarly designed studies should include more patients and controls. This further study should also address the question if age interferes with the metabolite concentrations obtained from microdialysis.

A strength of this explorative study is its comparative nature. Additionally, the rigorous application of inclusion and exclusion criteria built homogenous groups for further analyses. Different from previous studies [15, 20], the analysed metabolites were sampled not only during rest but also following exhaustive running activity and during recovery.

The question, if different biomarkers would have obtained significant effects when comparing CECS and control patients is open. Until now, there are no data available to answer this question. In a next step, pain mediators like prostaglandins could be a target for microdialysis studies. For further CECS microdialysis investigations, we recommend to develop a device to simultaneously measure the intra-compartmental pressure.

## Conclusions

In summary, our microdialysis study detected that lactate, glutamate, and glycerol concentrations in the extracellular fluid were increased for up to 20 min in both CECS and uninjured persons following exhaustive exercise. Glucose concentration was not compromised in CECS patients and control persons at all measured intervals. Our results indicate no specific involvement of these metabolites in the CECS pathogenesis. From a clinical point of view, microdialysis, as applied in this study, is not a candidate approach for diagnosing CECS.

## Data Availability

The datasets generated and/or analysed during the current study are not publicly available but are available from the corresponding author on reasonable request.

## Abbreviations

CECS: Chronic Exertional Compartment Syndrome
ANOVA: Analysis of Variance

## References

[1] Padhiar N, Thompson D, Padhiar C, Lohrer H. Podiatric Sports Medicine. In: Burrow JG, Rome K, Padhiar N, editors. Neale's Disorders of the Foot and Ankle. Edinburgh: Elsevier; 2020. p. 339–83.

[2] Lohrer H, Malliaropoulos N, Korakakis V, Padhiar N (2019). Exercise-induced leg pain in athletes: diagnostic, assessment, and management strategies. Phys Sportsmed.

[3] Styf J (2004). Compartment syndromes: diagnosis, treatment, and complications.

[4] Willy C, Gerngross H, Sterk J (1999). Measurement of intracompartmental pressure with use of a new electronic transducer-tipped catheter system. J Bone Joint Surg Am.

[5] Collinge C, Kuper M (2010). Comparison of three methods for measuring intracompartmental pressure in injured limbs of trauma patients. J Orthop Trauma.

[6] Roberts A, Franklyn-Miller A (2012). The validity of the diagnostic criteria used in chronic exertional compartment syndrome: a systematic review. Scand J Med Sci Sports.

[7] Turnipseed WD (2004). Clinical review of patients treated for atypical claudication: a 28-year experience. J VascSurg.

[8] Lohrer H, Nauck T (2007). Endoscopically assisted release for exertional compartment syndromes of the lower leg. Arch Orthop Trauma Surg.

[9] Verleisdonk EJ, Schmitz RF, van der Werken C (2004). Long-term results of fasciotomy of the anterior compartment in patients with exercise-induced pain in the lower leg. Int J Sports Med.

[10] Aweid O, Del BA, Malliaras P, Iqbal H, Morrissey D, Maffulli N, Padhiar N (2012). Systematic review and recommendations for intracompartmental pressure monitoring in diagnosing chronic exertional compartment syndrome of the leg. Clin J Sport Med.

[11] van den Brand JG, Nelson T, Verleisdonk EJ, van der Werken C (2005). The diagnostic value of intracompartmental pressure measurement, magnetic resonance imaging, and near-infrared spectroscopy in chronic exertional compartment syndrome: a prospective study in 50 patients. Am J Sports Med.

[12] Mohler LR, Styf JR, Pedowitz RA, Hargens AR, Gershuni DH (1997). Intramuscular deoxygenation during exercise in patients who have chronic anterior compartment syndrome of the leg. J Bone Joint Surg Am.

[13] Korth U, Klein J (2001). Methods and applications of microdialysis. Anaesthesiol lntens.

[14] Shippenberg TS, Thompson AC. Overview of microdialysis. Curr Protoc Neurosci. Chapter 7: Unit7; 2001. 10.1002/0471142301.ns0701s00 PMC2538639 18428520

[15] Korth U, Merkel G, Fernandez F, Jandewerth O, Dogan G, Koch T, van Ackern K, Weichel O, Klein J (2000). Tourniquet-induced changes of energy metabolism in human skeletal muscle monitored by microdialysis. Anesthesiology.

[16] Rutten EP, Engelen MP, Schols AM, Deutz NE (2005). Skeletal muscle glutamate metabolism in health and disease: state of the art. Curr Opin Clin Nutr Metab Care.

[17] Kjaer M, Langberg H, Bojsen-Moller J, Koskinen SO, Mackey A, Heinemeier K, Holm L, Skovgaard D, Dossing S, Hansen M, Hansen P, Haraldsson B, Caroe I, Magnusson SP (2008). Novel methods for tendon investigations. Disabil Rehabil..

[18] Langberg H, Skovgaard D, Karamouzis M, Bulow J, Kjaer M (1999). Metabolism and inflammatory mediators in the peritendinous space measured by microdialysis during intermittent isometric exercise in humans. J Physiol.

[19] Alfredson H, Thorsen K, Lorentzon R (1999). In situ microdialysis in tendon tissue: high levels of glutamate, but not prostaglandin E2 in chronic Achilles tendon pain. Knee Surg Sports Traumatol Arthrosc.

[20] Alfredson H, Ljung BO, Thorsen K, Lorentzon R (2000). In vivo investigation of ECRB tendons with microdialysis technique--no signs of inflammation but high amounts of glutamate in tennis elbow. Acta Orthop Scand.

[21] Derr J (2006). Valid paired data designs: make full use of the data without "double-dipping". J Orthop Sports Phys Ther.

[22] Moher D, Schulz KF, Altman DG (2001). The CONSORT statement: revised recommendations for improving the quality of reports of parallel-group randomised trials. Lancet..

[23] Motulsky H. Intuitive biostatistics. Oxford University Press Inc 2018.

[24] Wallensten R, Eklund B (1983). Intramuscular pressures and muscle metabolism after short-term and long-term exercise. Int J Sports Med.

[25] Qvarfordt P, Christenson JT, Eklof B, Ohlin P, Saltin B (1983). Intramuscular pressure, muscle blood flow, and skeletal muscle metabolism in chronic anterior tibial compartment syndrome. Clin Orthop Relat Res.

[26] Edmundsson D, Toolanen G, Thornell LE, Stal P (2010). Evidence for low muscle capillary supply as a pathogenic factor in chronic compartment syndrome. Scand J Med Sci Sports.

[27] Dickenson AH, Chapman V, Green GM (1997). The pharmacology of excitatory and inhibitory amino acid-mediated events in the transmission and modulation of pain in the spinal cord. Gen Pharmacol.

[28] Tucker AK (2010). Chronic exertional compartment syndrome of the leg. Curr Rev Musculoskelet Med.

[29] Franklyn-Miller A, Roberts A, Hulse D, Foster J (2014). Biomechanical overload syndrome: defining a new diagnosis. Br J Sports Med.

[30] Orlin JR, Oen J, Andersen JR (2013). Changes in leg pain after bilateral fasciotomy to treat chronic compartment syndrome: a case series study. J Orthop Surg Res.

[31] Burrus MT, Werner BC, Starman JS, Gwathmey FW, Carson EW, Wilder RP, Diduch DR (2015). Chronic leg pain in athletes. Am J Sports Med.

[32] Moen MH, Tol JL, Weir A, Steunebrink M, De Winter TC (2009). Medial tibial stress syndrome: a critical review. Sports Med.

[33] Stickley CD, Hetzler RK, Kimura IF, Lozanoff S (2009). Crural fascia and muscle origins related to medial tibial stress syndrome symptom location. Med Sci Sports Exerc.

